# Variability in the Chemical Composition and In Vitro Ruminal Fermentation of Olive Cake By-Products

**DOI:** 10.3390/ani9030109

**Published:** 2019-03-22

**Authors:** Carlos N. Marcos, Paloma García-Rebollar, Carlos de Blas, María Dolores Carro

**Affiliations:** Departamento de Producción Agraria, Escuela Técnica Superior de Ingeniería Agronómica, Agroalimentaria y de Biosistemas, Universidad Politécnica de Madrid, Ciudad Universitaria, 28040 Madrid, Spain; navarro-88@hotmail.com (C.N.M.); paloma.grebollar@upm.es (P.G.-R.); c.deblas@upm.es (C.d.B.)

**Keywords:** olive cake, storage time, processing, in vitro ruminal fermentation, prediction models

## Abstract

**Simple Summary:**

Olive cake is a by-product of oil production that can be used in ruminant feeding, but its composition can vary with multiple factors. The objective of this study was to determine the variability in the chemical composition and in vitro ruminal fermentation of 42 olive cake samples to evaluate their nutritive value for ruminants. The results showed that the chemical composition of olive cake is highly variable and is markedly affected by processing type and its storage time before processing. Cyclone olive cake had greater nutritive value for ruminants than both crude and extracted olive cake. All olive cake types were poorly degraded in vitro and, therefore, their nutritive value is highly dependent on their ether extract content. Statistical models using olive cake processing type, and either the chemical composition or storage time before processing as covariates, can be used in the practice to predict in vitro ruminal fermentation variables of olive cake samples. The obtained information may contribute to increase the use of olive cake in ruminant feeding.

**Abstract:**

The objective of this study was to determine the variability in the chemical composition and in vitro ruminal fermentation of olive cake (OC) by-products. Forty-two OC samples with different storage times (1–14 months) and processing (25 crude (COC), 9 exhausted (EOC) and 9 cyclone (CYOC)) were fermented in vitro with sheep ruminal fluid. Exhausted OC samples had a lower ether extract content than COC and CYOC (15.9, 110 and 157 g/kg dry matter (DM), respectively), but greater neutral detergent fiber (NDF; 645, 570 and 441 g/kg DM) and acid insoluble nitrogen (9.76, 8.10 and 8.05 g/kg DM) content. Exhausted OC had the greatest (*p* < 0.05) average gas production rate (AGPR), whereas the greatest fermented organic matter (FOM) was obtained for EOC and CYOC. The best single predictor of the AGPR was total sugars content (*R*^2^ = 0.898), whereas NDF was the best one for FOM (*R*^2^ = 0.767; *p* < 0.001). Statistical models using storage time as a predictor variable had lower accuracy and *R*^2^ values than those from the chemical composition. In summary, the nutritive value of OC was highly dependent on its processing, but its ether extract content did not negatively affect ruminal fermentation parameters, which could be estimated from either carbohydrate composition or storage time.

## 1. Introduction

The use of agroindustrial by-products in animal feeding is increasing worldwide, as it is a viable solution to value wastes and minimize environmental pollution from food industries [1]. However, the chemical composition of these ingredients is highly variable and frequently only limited data on their nutritive value are available, thus limiting their use as regular ingredients in animal rations.

The Mediterranean area concentrates most of world olive oil production, with Spain being the world’s leading producer [2]. The system of olive oil extraction most widely used in Spain generates a wet solid-waste (500–700 g moisture/kg) called “alperujo”, which is stored in open-air ponds before being processed to obtain different types of olive cake (OC). After removal of olive stones, the alperujo is dried to produce crude olive cake (COC), which can be treated with solvents to extract the residual oil (pomace olive oil), obtaining the exhausted olive cake (EOC). In order to clean the air used to dry the alperujo before being emitted to the atmosphere, the OC particles floating in the air are collected from cyclone decanters generating cyclone OC (CYOC). Crude OC is the by-product more frequently used for ruminant feeding, whereas EOC is mostly used as biomass fuel for electricity generation, and small amounts of CYOC are marketed as high-quality COC. The chemical composition of OC by-products can be influenced by multiple factors such as the olive cultivar, the ripeness of the fruit, the alperujo storage conditions and the processing techniques [3,4]. The amount of data on the chemical composition of different types of OC is variable [5], but to the best of our knowledge there is no information on the composition of CYOC. Moreover, the existing information on the dry matter ruminal degradability of OC is scarce, although available data indicate low values with a wide range of variation (from 8% to 42%) [3]. Therefore, the first objective of this study was to analyse the variability in the chemical composition and in vitro ruminal fermentation of different types of OC to evaluate their potential nutritive value for ruminants. A previous study [6] showed a marked influence of the storage time of the alperujo on the chemical composition and in vitro fermentation of OC and, therefore, samples of OC stored for different times were obtained at various extraction plants. The samples used in the study of Marcos et al. [6] were included in the present study to increase the sample size. In addition, statistical models were developed to estimate the fermentation characteristics of OC from processing type and either the chemical composition or storage time of the alperujo in the ponds.

## 2. Materials and Methods

### 2.1. Olive Cake Samples

In order to obtain OC samples representative of those available in Spain for animal feeding, a total of 42 samples of OC were obtained at nine different extraction plants located in the South of Spain during the 2015/16 and 2016/17 olive oil campaigns. Samples were classified according to processing type (COC, EOC and CYOC) and storage time in the open-air ponds (from 1 to 14 months). Twenty-five samples of COC were obtained by drying the alperujo and all of them were pelleted, whereas 9 EOC samples were generated by subjecting the dried alperujo to oil extraction using hexane and drying the exhausted cake, and 8 CYOC samples came from the particles collected from the air in cyclone decanters during the alperujo drying process. Processing of all OC samples were performed at the extraction plants using the habitual procedures in each of them. Storage time of the alperujo in the ponds was 3 months or lower for 10 samples (4 COC, 3 EOC and 3 CYOC), between 4 and 6 months for 16 samples (11 COC, 3 EOC and 2 CYOC), and 7 months or longer for 16 samples (10 COC, 2 EOC and 4 CYOC. All samples were ground to pass a 1-mm screen before chemical analyses and in vitro incubations.

### 2.2. In Vitro Incubations

All procedures involving animals were approved by the Institutional Animal Care and Use Committee of the Comunidad Autónoma de Madrid (PROEX 035/17). Four Lacaune sheep (64.7 ± 2.10 kg body weight), each provided with a permanent cannula in the rumen, were used as donors of ruminal fluid for the in vitro incubations. Sheep were individually housed, had access to fresh water over the trial, and were fed a diet composed of grass hay and concentrate in a 2:1 ratio at a rate of 45 g dry matter (DM)/kg body weight ^0.75^. Diet was fed in two equal meals at 08:00 and 18:00 h. The diet contained 112, 350 and 166 g of crude protein, neutral detergent fiber (NDF) and acid detergent fiber (ADF) per kg DM, respectively.

Two in vitro trials were conducted in different days using the same methodology to determine gas production kinetics and the main ruminal fermentation parameters, respectively. In each incubation trial, four replicates per OC sample were obtained by conducting the incubations on four different days and each day the ruminal fluid from one sheep was used as the inoculum. Ruminal contents of each sheep were obtained before the morning feeding, strained through four layers of cheesecloth and immediately transported to the laboratory in thermal flasks. The fluid was mixed with the culture medium [7] (without trypticase; 39 °C) in a 1:4 ratio under CO_2_ flushing. Samples (200 mg of DM) of each OC were weighed into 60-mL vials before the addition of 20 mL of the mixture of ruminal fluid and culture medium. Vials were then sealed with rubber stoppers and incubated at 39 °C. In the first trial, to determine gas production kinetics, vials were incubated for 120 h, and gas production was measured at 3, 6, 9, 12, 15, 22, 26, 31, 36, 48, 58, 72, 96 and 120 h using a pressure transducer (Widereager Wide Range Pressure Meter, Sper Scientific LTD, Scottsdale, AZ, USA) and a plastic syringe. The gas produced at each measurement time was released to prevent gas accumulation. Blanks (vials without a sample; two per inoculum) were also incubated to correct values of gas production for endogenous gas production. The second incubation trial was conducted to analyze the main fermentation parameters after 24 h of incubation. Gas production was measured as described before. Vials were then uncapped, their content was homogenized and the pH was measured using a Crison Basic 20 pH meter (Crison Instruments, Barcelona, Spain). For determining volatile fatty acid (VFA) and NH_3_–N concentrations, 3 mL of the content of each vial was mixed with 3 mL of HCl 0.5 N, and samples were stored at −20 °C until analyses.

In order to calculate DM effective degradability (DMED), the potential in vitro DM degradability (DMD_120_) was determined by weighing, in triplicate, 300 mg of each OC sample into polyester bags (30-µm pore size; Ankom Corp #57, Ankom Technology Corp., Fairport, NY, USA). Bags were incubated with a 1:4 ratio of ruminal fluid (mixture of the fluid from the 4 sheep) to incubation medium [7] in an Ankom Daisy II incubator (Ankom Technology Corp., Fairport, NY, USA) at 39 °C and under continuous rotation. After 120 h, bags were washed with cold water, dried at 60 °C for 48 h, and weighted to calculate DMD_120_.

### 2.3. Chemical Analyses

The proximate composition of OC samples was analyzed using the procedures of the Association of Official Agricultural Chemists [8] for dry matter (ID 934.01), ash (ID 048.13) and ether extract (EE; ID 945.16). Concentrations of NDF, ADF and acid detergent lignin (ADL) were determined following the procedures of Van Soest et al. [9] and Robertson and Van Soest [10], respectively, using an ANKOM220 Fiber Analyzer unit (ANKOM Technology Corporation, Fairport, NY, USA). Sodium sulphite was used in the sequential analysis of NDF, ADF and ADL, and results were expressed exclusive of residual ash. Nitrogen was measured by the Dumas combustion method employing a Leco FP258 Nitrogen Analyzer (Leco Corporation, St. Joseph, MI, USA). Acid detergent insoluble nitrogen (ADIN) was determined by analyzing the N content in the residue obtained after treatment with acid detergent solution. Total sugars and total soluble polyphenols (TSP) were analyzed by colorimetric methods following the anthrone method [11] and the Folin–Ciocalteu assay [12], respectively, using an Epoch spectrophotometer (BioTek Instruents Inc., Winooski, VT, USA).

Concentrations of NH_3_–N in vial contents after 24 h of fermentation were determined by the phenol-hypochlorite method as described by Weatherburn [13], and those of VFA were conducted by gas chromatography as described by García-Martínez et al. [14]. All analyses were performed in duplicate.

### 2.4. Calculations and Statistical Analyses

Data of gas production were fitted with time using the model: Gas = PGP (1 − e ^(−c (t −^ *^lag)^*^)^), where PGP is the potential gas production (mL), *c* is the fractional rate of gas production (h^−1^), *lag* is the initial delay in the onset of gas production (h), and t is the time of gas measurement. Gas production parameters were estimated using the NLIN procedure of SAS [15] by an iterative least squares procedure. The average gas production rate (AGPR; mL gas/h) was defined as the average gas production rate between the start of the incubation and the time when half of the PGP was reached, and was calculated as AGPR = PGP *c/*[2 (ln2 + *c lag*)]. The DMED was estimated for a rumen particulate outflow (*K*p) of 0.042 per h from gas production parameters and DMD_120_ as: DMED = [(DMD_120_ × *c*)/(*c* + *K*p)] e ^(^^−^*^c^* ^× lag)^. This rumen particulate outflow corresponds to a rumen retention time of 24 h. Finally, the amount of fermented organic matter (FOM) was calculated from acetate, propionate and butyrate production in each vial as described by Demeyer [16].

The chemical composition data of OC samples was analyzed using the PROC GLM of SAS [15] with OC type as the main effect. Data on fermentation parameters were analyzed as a mixed model using the PROC MIXED of SAS [15], where the effect of OC type (COC, EOC and CYOC) was considered fixed and inoculum was considered a random effect. In both models, when effects were significant, means were compared by using the Tukey’s test. Pearson correlation coefficients among the chemical composition of OC samples, gas production or fermentation parameters, and storage time of the alperujo were assessed using the PROC CORR of SAS [15]. Models to predict the AGPR and FOM were developed using the PROC GLM of SAS, including OC type (COC, EOC and CYOC) as the fixed effect and chemical fractions as linear and quadratic covariates. The interaction between OC type and the chemical composition was also studied. Slopes of the models for different OC types were compared by Student’s test. In addition, the accuracy of predicting the AGPR and FOM from OC type and the duration (months) of the alperujo storage in ponds as a covariate was evaluated using the same model.

## 3. Results

### 3.1. Influence of Olive Cake Processing Type on Chemical Composition and In Vitro Fermentation

The chemical composition of OC samples (Table 1) was highly variable. There were great differences in EE content (coefficient of variation (CV) of 53%), but TSP and sugars were the most variable fractions, with CV values ranging from 44.1% to 126%. For each of the three OC types, the most variable fractions were also sugars (CV ≥ 107%) and TSP (CV ≥ 41.1%), whereas CV values for the rest of the chemical fractions were lower than 24%, with the exception of EE for EOC (69.7%). Crude OC and EOC groups showed similar CV values for most components analyzed, and CYOC had greater CV values for ADL and ADIN compared to COC and EOC.

As shown in Table 2, CYOC had greater (*p* < 0.05) DM, N and EE content than COC, but lower organic matter (OM), NDF, ADF and ADL content. Compared with COC, EOC had lower EE but greater NDF, ADIN and TSP content. Total sugars were the only fraction analyzed in which no differences among OC types were observed.

Samples of COC had lower (*p* < 0.05) PGP, AGPR and DMED values than EOC, whereas CYOC showed intermediate values of PGP and DMED (Table 3). After 24 h of incubation, neither total VFA production nor molar proportions of individual VFA differed among OC types, with the exception of minor VFA proportions that were lower (*p* = 0.003) for EOC compared with COC samples. Samples of EOC also had lower NH_3_–N concentrations than both COC and CYOC. Calculated FOM values were greater for EOC and CYOC samples than for COC.

### 3.2. Estimation of Fermentation Parameters

The average gas production rate and FOM were selected for developing prediction models as they reflect the fermentation rate and extent of rumen fermentation, respectively. Table 4 shows the correlation coefficients between the chemical composition, fermentation parameters (AGPR and FOM) and storage time of the alperujo. Sugars and TSP were the chemical fractions more closely correlated with the AGPR (r = 0.90 and 0.69, respectively; *p* < 0.001), whereas NDF, total sugars and TSP content were highly correlated (r = −0.68, 0.82 and 0.78, respectively; *p* < 0.001) with FOM. Storage time showed negative correlations (r ≥ −0.74; *p* < 0.001) with total sugars and TSP content, and both fermentation parameters.

The prediction equations to estimate the AGPR and FOM from OC type and chemical composition are shown in Table 5. The total sugars content was the best covariate to predict the AGPR, as the models including TSP content or any other chemical fractions as predictor variables had greater residual standard deviation (RSD) values (i.e., 0.68 mL/h for TSP content; equations not shown). The sugars content had a positive linear effect (*p* < 0.001) and a quadratic negative effect (*p* < 0.001) on the AGPR. Moreover, an OC type effect was detected (*p* < 0.001) on the intercept term, which had the lowest value in the CYOC model and the greatest in the EOC model.

The NDF content was the best predictor variable of FOM, as models including either sugars or TSP content had greater RSD values (32.1 and 35.0 g/kg OM, respectively; equations not shown) than the model using NDF (27.2 g/kg OM). The NDF content had the same negative effect on FOM values for all OC types, as no OC type–NDF interaction was detected on the slope of the model. The type of OC affected (*p* = 0.031) the intercept term, which had the lowest value in the CYOC model and the greatest in the EOC model.

The storage time was closely correlated (*p* < 0.001) with sugars and TSP content (Table 4) and, therefore, prediction models of fermentation parameters including storage time were developed separately from those involving chemical analyses. As shown in Table 5, the prediction equations of the AGPR and FOM with OC type and storage time as a covariate had lower R^2^ and greater RSD values than those using OC type and chemical fractions. A linear and quadratic effect of storage time on the AGPR and FOM was observed, but the interaction between OC type and storage time (*p* < 0.001) on the slope of the AGPR model indicates that the effect of storage time differed among OC types. For a fixed increment of time, the AGPR increased in the order CYOC ≤ COC < EOC. In contrast, neither the intercept nor the slope of the model for FOM prediction was affected by OC processing type.

## 4. Discussion

### 4.1. Influence of Olive Cake Processing on Chemical Composition and In Vitro Fermentation

The chemical composition of COC and EOC samples was in agreement with values reported by others [3,5,17], and both OC types showed high variability. Factors such as the olive cultivar, ripeness of the fruit, agronomic conditions, storage time in open-air ponds and climatic conditions influence the chemical composition of the alperujo [4] and, therefore, the composition of the OC obtained. As expected, EE content was lower in EOC than in COC due to the second oil extraction and this caused an increase in the remaining chemical components, although only NDF showed significant differences. The degree of lignification of NDF and ADF fractions (36% and 50%, respectively; calculated as (lignin/NDF) × 100) was similar between OC types reflecting no changes due to processing. All OC samples had low N content (≤23.5 g/kg DM), and in accordance with others [3,5] ADIN was a high proportion of total N (51.3, 54.8 and 44.7% for COC, EOC and CYOC, respectively), indicating low N availability. However, NH_3_–N concentrations in the in vitro incubations were above the level limiting in vitro ruminal microbial growth [18] for all OC samples due to the use of an N-enriched medium. The greater ADIN concentrations in EOC compared with COC and CYOC samples were attributed to the second heat treatment applied to remove the hexane solvent used in the extraction of pomace olive oil from COC, as heating may lead to the formation of indigestible compounds via Maillard reactions between sugar aldehyde groups and free amino groups [19].

To the best of our knowledge, there was no previous information on the chemical composition of CYOC in the literature. The lower NDF and ADF content of CYOC than in COC and EOC may reflect the smaller amount of olive stone fragments, as CYOC is composed of the lighter pomace particles floating in the air collected during drying the alperujo. The lower NDF, ADF and ADL content of CYOC compared with both COC and EOC also supports this hypothesis, as hemicellulose, cellulose and lignin are the main components of olive stones [20]. In addition, CYOC had the greatest EE content and, therefore, it is expected to have greater energy content for ruminants than COC and EOC.

In agreement with the results reported by others (reviewed by Molina-Alcaide and Yáñez Ruiz, [3]) the ruminal degradability of OC was low, but there was a wide variability in DMED values which ranged from 99.2 to 378 g/kg DM. For example, Álvarez-Rodríguez et al. [21] observed that only 120 g/kg DM of a sample of EOC disappeared from the rumen after 72 h of incubation, whereas this value was 370 g/kg DM for a COC sample, and a slightly greater in situ value (420 g/kg DM) was reported by Martín-García et al. [22] for a COC sample. Variations in DMED could be partly explained by the chemical composition of OC, as DMED was positively correlated with total sugars (*p* < 0.001) and negatively with NDF, ADF and ADL (*p* ≤ 0.024; values not shown) content.

Both the unchanged VFA production and VFA profile in all OC groups indicate a similar fermentation pattern. Unsaturated free fatty acids resulting from the triglyceride hydrolysis are toxic to fibrolytic bacteria [23], but the similar acetate and butyrate proportions observed in the three OC groups does not indicate a negative effect of the high EE content of some COC and CYOC samples on fiber fermentation. A maximal level of 60 g of fat per kg of DM is usually recommended in the diet of ruminants to avoid reductions in fiber digestibility [24]; other studies revealed no negative effect of greater EE levels both in vivo [25] and in vitro [26]. Finally, the lack of correlation between EE and any fermentation parameter supports the idea that there was no negative effect of the high EE content on ruminal fermentation.

The hydrolysis of olive oil lipids has been reported to be much lower than that of more unsaturated oils such as linseed oil (68% vs. 95% at 24-h incubation, respectively) [27], thus liberating less fatty acids. In addition, the high lignification degree of the NDF in all OC types (≥34.9%) indicates low NDF degradability and this may help to explain the lack of an apparent negative effect of EE content on total VFA production from COC and CYOC samples. In contrast, the lower the AGPR observed for COC and CYOC compared with EOC might indicate a reduced fermentation. However, gas production data from samples with high EE content should be interpreted with caution, as no gas arises from EE fermentation in the rumen, and total gas production can even be reduced by fat supplementation due to decreased CH_4_ production [28].

### 4.2. Estimation of Fermentation Parameters

The average gas production rate was influenced by the OC processing type and the intercept of the model was greater for EOC than for COC and CYOC, possibly due to the differences in their EE content, and, therefore, in the amount of potentially fermentable dry matter, as no gas is formed from fat fermentation. The positive linear effect of sugars content in the prediction equations of the AGPR reflects that sugars contribute to increased gas production in the first hours of incubation, when sugars are rapidly and extensively fermented by rumen microorganisms. In fact, sugars/EE ratio was greater in EOC than in COC and CYOC samples (6.8, 0.31 and 0.28, respectively). However, there was also a negative quadratic effect of sugars content that might be due to the positive relationship observed between sugars and TSP content in OC samples. The presence of TSP in feeds can reduce feed ruminal degradability [29], and negative correlations between TSP content and DM degradability are frequently reported [30].

The significant effect of OC type on the intercept of the FOM model indicates differences between OC types, with COC and CYOC having lower FOM values than EOC for samples with similar NDF content. This might be related to the greater EE content in COC and CYOC, as EE was not related to NDF content and EE does not lead to VFA production (only low VFA amounts are generated from glycerol fermentation [31]). The lack of influence of OC type on the slope of the FOM prediction model reflects similar changes in FOM with increases in NDF for all OC types, as clearly shown by the similar slopes observed in the linear models developed individually for each OC group (r = −0.622, −0.648 and −0.690 for COC, EOC and CYOC samples, respectively). This is consistent with the similar lignification degree of the NDF in all OC types (36.0%, 34.9% and 37.0% for COC, EOC and CYOC, respectively), as lignin is one of the major factors limiting NDF degradability.

Models involving OC type and storage time as predictor variables were also developed, as this would be a cheap and rapid form to estimate fermentation parameters. Differences in intercept values on the AGPR model were related to the differences in EE content among OC types that were not correlated with changes in the covariate of the model. The negative effect of storage time on the slope is in accordance with the observed changes in the chemical composition of OC over the storage period. Sugars content in OC samples clearly decreased as storage time in the pond augmented (r = −0.769; *p* < 0.001; *n* = 42), whereas NDF content, a less fermentable fraction, increased with advancing storage time (r = 0.395; *p* = 0.010; *n* = 42).

The significant interaction between OC type and storage time on the slope observed in the AGPR model reflects a different timely evolution for each OC type. The greater slope of the EOC model compared with those for COC and CYOC samples may reflect the greater increases in NDF fraction with time (167 g/kg DM from months 1 to 8) in EOC compared with those in COC and CYOC (109 and 87 g/kg DM, respectively), because NDF fermentability is lower than in other fractions (i.e., sugars, starch, etc.). In contrast, in the prediction of FOM, there was no effect of OC type on the intercept, which is consistent with the lack of correlation between EE and storage time. The negative effect of storage time on the slope of the FOM model reflected the decrease in total sugars and, therefore, in OM fermentation as the alperujo storage time advanced. Finally, the quadratic effect of storage time in all models confirms the results of Marcos et al. [6], who observed that the loss of the nutritive value of OC was more pronounced during the first months of storage and almost imperceptible from 6 storage months.

Although the models using OC type and storage time as predictor variables had lower R^2^ and greater RSD values than those involving OC type and the chemical composition, it has to be taken into account that analysis of the chemical composition is expensive and time consuming. The possibility of predicting in vitro fermentation parameters from OC type and storage time could be a cheap and rapid alternative.

## 5. Conclusions

The results show that the chemical composition of OC by-products is highly variable, markedly affected by the processing type and storage time of the alperujo before processing. However, all samples had high lignification of both NDF and ADF fractions and low N content, with ADIN being greater than 44% of total N. Cyclone OC had the lowest NDF and the greatest EE content, as well as an intermediate sugars content, which indicates that it would have greater nutritive value for ruminants than COC and EOC. All OC types were poorly degraded in vitro and, therefore, their nutritive value for ruminants is highly dependent on their EE content. Models using OC processing type, and either the chemical composition or the alperujo storage time before processing as covariates, were useful to predict the in vitro ruminal fermentation variables of OC.

## Figures and Tables

**Table 1:** Chemical composition (g/kg dry matter unless stated otherwise) of olive cake (OC) samples (*n* = 42).

Item	Mean	CV	OC Type (Range of Values and CV) ^1^		
			Crude OC (*n* = 25)	Exhausted OC (*n* = 9)	Cyclone OC (*n* = 8)
Dry matter (g/kg)	906	2.77	862–946 [2.49]	860–908 [1.95]	914–952 [1.43]
Organic matter	913	1.98	873–948 [2.03]	894–935 [1.38]	885–910 [0.91]
Neutral detergent fiber	561	17.8	402–721 [13.4]	528–766 [14.0]	367–528 [13.5]
Acid detergent fiber	409	17.2	298–501 [12.6]	385–533 [12.2]	256–374 [13.5]
Acid detergent lignin	201	14.6	170–250 [10.0]	197–261 [9.41]	123–190 [15.4]
Nitrogen (N)	16.7	14.1	13.1–18.6 [10.7]	15.8–23.5 [13.0]	12.5–22.2 [17.5]
Acid detergent insoluble N	8.45	19.8	5.20–11.9 [18.4]	7.85–12.2 [14.4]	4.70–10.8 [23.9]
Ether extract	99.0	53.1	73.3–145 [21.3]	4.00–32.0 [69.7]	108–195 [20.0]
Total sugars	43.2	123	7.10–202 [126]	8.00–197 [107]	6.50–149 [120]
Total soluble polyphenols	14.5	44.8	5.30–29.1 [42.0]	8.60–29.2 [41.1]	9.70–29.2 [41.9]

^1^ Minimum and maximum values and coefficients of variation (CV; %). Crude OC: samples were dried and pelletized; Exhausted OC: samples were dried and subjected to a second oil extraction using hexane; Cyclone OC: samples were obtained from cyclone decanters during drying COC. *n* = number of samples. Values between brackets are CV (%).

**Table 2:** Influence of olive cake (OC) processing on chemical composition (g/kg dry matter unless stated otherwise).

Item	OC Type ^1^			SEM ^2^	*p-*Value
	Crude OC (*n* = 25)	Exhausted OC (*n* = 9)	Cyclone OC (*n* = 8)		
Dry matter (g/kg)	904 ^a^	886 ^a^	934 ^b^	6.1	<0.001
Organic matter	916 ^b^	919 ^b^	895 ^a^	4.9	0.004
Neutral detergent fiber	570 ^b^	645 ^c^	441 ^a^	23.4	<0.001
Acid detergent fiber	409 ^b^	454 ^b^	309 ^a^	15.4	<0.001
Acid detergent lignin	205 ^b^	225 ^b^	163 ^a^	6.5	<0.001
Nitrogen (N)	15.8 ^a^	17.8 ^ab^	18.0 ^b^	0.65	0.013
Acid detergent insoluble N	8.10 ^a^	9.76 ^b^	8.05 ^a^	0.473	0.025
Ether extract ^3^	110 ^b^	15.9 ^a^	157 ^c^	7.35	<0.001
Total sugars ^3^	33.9	57.8	43.9	11.0	0.244
Total soluble polyphenols ^3^	12.4 ^a^	18.4 ^b^	16.7 ^ab^	1.93	0.031

^a,b^ Within each chemical fraction, mean values with different superscripts differ (*p* < 0.05). ^1^ Crude OC: samples were dried and pelletized; Exhausted OC: samples were dried and subjected to a second oil extraction using hexane; Cyclone OC: samples were obtained from cyclone decanters during drying COC. *n* = number of samples. ^2^ SEM = Standard error of the mean. ^3^ the Kruskal–Wallis test was used to compare these variables, as they were not normally distributed.

**Table 3:** Influence of olive cake (OC) processing on gas production parameters, dry matter effective degradability (DMED) and fermentation parameters in a 24-h in vitro fermentation of OC samples.

Item	OC Type ^1^			SEM ^2^	*p-*Value
	Crude OC (*n* = 25)	Exhausted OC (*n* = 9)	Cyclone OC (*n* = 8)		
Gas production parameters ^2^					
PGP (mL/g dry matter)	64.5 ^a^	78.1 ^b^	74.4 ^ab^	3.67	0.008
*c* (h^−1^)	0.048 ^b^	0.054 ^b^	0.040 ^a^	0.0022	<0.001
*Lag* (h)	0.16	0.21	0.33	0.091	0.208
AGPR (mL/h)	2.23 ^a^	3.07 ^b^	2.13 ^a^	0.182	<0.001
DMED (g/kg DM)	208 ^a^	245 ^b^	217 ^ab^	10.1	0.025
Fermentation parameters					
Total volatile fatty acids (VFA; mmol/g DM)	3.35	3.57	3.56	0.090	0.067
Molar proportions of VFA (mol/100 mol)					
Acetate (Ac)	61.8	62.1	62.2	0.24	0.351
Propionate (Pr)	21.4	21.7	21.6	0.24	0.642
Butyrate	11.0	11.1	10.7	0.15	0.263
Minor VFA ^3^	5.79 ^b^	5.16 ^a^	5.46 ^ab^	0.15	0.003
Ac/Pr (mol/mol)	2.92	2.89	2.91	0.036	0.854
NH_3_–N (mg/L)	222 ^b^	207 ^a^	222 ^b^	6.3	0.004
FOM (g/kg organic matter) ^4^	311 ^a^	333 ^b^	338 ^b^	8.6	0.025

^a,b^ Within each parameter, mean values with different superscripts differ (*p* < 0.05). ^1^ Crude OC: samples were dried and pelletized; Exhausted OC: samples were dried and subjected to a second oil extraction using hexane; Cyclone OC: samples were obtained from cyclone decanters during drying COC. *n* = number of samples. ^2^ PGP: potential gas production; *c*: fractional rate of gas production; *Lag*: time until the production of gas begins; AGPR: the average gas production rate until half of the PGP has been reached; FOM: fermented organic matter, DMED was calculated for a rumen passage rate of 0.042 per h. ^3^ Calculated as the sum of isobutyrate, isovalerate and valerate. ^4^ Fermented organic matter calculated from VFA production as described by Demeyer (1991).

**Table 4:** Correlation matrix (Pearson coefficient and *p* values in brackets; *n* = 42) of the chemical composition of olive cake (OC) samples and storage time with the average gas production rate (AGPR) and fermented organic matter (FOM) of OC samples measured in 24-h in vitro incubations (only *p* < 0.05 values are shown) ^1^.

Item	Neutral Detergent Fiber	Acid Detergent Fiber	Acid Detergent Lignin	*N*	Acid Detergent Insoluble N	Ether Extract	Total Sugars	Total Soluble Polyphenols	Storage Time
Neutral detergent fiber	-	0.99 (<0.001)	0.90 (<0.001)	-	0.43 (0.004)	−0.61 (<0.001)	−0.43 (0.004)	−0.47 (0.002)	0.39 (0.010)
Acid detergent fiber	-	-	0.93 (<0.001)	-	0.44 (0.003)	−0.63 (<0.001)	−0.40 (0.009)	−0.44 (0.003)	0.36 (0.018)
Acid detergent lignin	-	-	-	-	0.49 (0.001)	−0.61 (<0.001)	−0.34 (0.027)	−0.41 (0.007)	0.36 (0.018)
N	-	-	-	-	0.58 (<0.001)	-	−0.33 (0.035)	-	-
Acid detergent insoluble N	-	-	-	-	-	−0.42 (0.006)	-	-	-
Ether extract	-	-	-	-	-	-	-	-	-
Total sugars	-	-	-	-	-	-	-	0.60 (<0.001)	−0.75 (<0.001)
Total soluble polyphenols	-	-	-	-	-	-	-	-	−0.74 (<0.001)
Fermentation parameters									
AGPR	−0.35 (0.024)	−0.31 (0.048)	-	−0.32 (0.039)	-	-	0.90 (<0.001)	0.69 (<0.001)	−0.76 (<0.001)
FOM	−0.68 (<0.001)	−0.64 (<0.001)	−0.58 (<0.001)	-	-	-	0.82 (<0.001)	0.78 (<0.001)	−0.75 (<0.001)

^1^ AGPR: the average gas production rate until it reached half of PGP; fermented organic matter (FOM) was calculated from volatile fatty acids production as described by Demeyer (1991).

**Table 5:** Models for predicting the average gas production rate (AGPR; mL/h) and fermented organic matter (FOM; g/kg OM) after 24-h in vitro incubation of olive cake (OC) samples using OC type (crude (COC), exhausted (EOC) and cyclone (CYOC)) as the fixed effect and either chemical fractions or storage time as covariates (*n* = 42) ^1^.

Covariate	Parameter ^3^	Prediction Equation ^4^	RSD ^5^	*p-*Value ^6^				*R* ^2^
				*p*_OC_	*p*_L_	*p*_OC_ * _L_	*p*_Q_	
Chemical fractions (g/kg dry matter)	AGPR (mL/h)	(1.54 (±0.169) + **A**) + 0.039 (±0.0040) **Total sugars** − 0.00010 (±0.000021) **Total sugars ^2^** A values: −0.393 (±0.1601), 0 and −0.645 (±0.1982) for COC, EOC and CYOC, respectively	0.41	<0.001	<0.001	-	<0.001	0.898
Chemical fractions (g/kg dry matter)	FOM (g/kg organic matter)	(743 (±39.1) + **A**) − 0.64 (±0.059) **Neutral detergent fiber** A values: −69.1 (±11.84), 0 and −124 (±18.2) for COC, EOC and CYOC, respectively	27.2	0.031	<0.001	*-*	-	0.767
Storage time (months)	AGPR (mL/h)	(6.55 (±0.480) + **A**) + **B** * **Time** + 0.0355 (±0.00907) **Time ^2^** A values: −1.65 (±0.553), 0 and −2.56 (±0.663) for COC, EOC and CYOC, respectively B values: −0.71 (±0.122), −0.95 (±0.120) and −0.58 (±0.109) for COC, EOC and CYOC, respectively	0.55	0.002	-	<0.001	<0.001	0.796
Storage time (months)	FOM (g/kg organic matter)	432 (±17.4) − 27.2 (±5.91) * **Time** + 1.07 (±0.481) **Time ^2^**	35.7	<0.001	-	-	0.019	0.618

^1^ COC samples were dried and pelletized; EOC samples were dried and subjected to an extraction using hexane; CYOC samples were obtained from a cyclone separator after drying the crude samples. ^3^ AGPR: the average gas production rate until it reached half of PGP; FOM: fermented organic matter calculated from volatile fatty acids production as described by Demeyer (1991). ^4^ Values in parentheses are standard errors. ^5^ Residual standard deviation. ^6^ *p*_OC_: effect of OC type; *p*_L_: linear effect of covariate; *p*_Q_: quadratic effect of covariate; *p*_OC_ * _L_: OC type–covariate interaction effect.
